# Metastatic Renal Cell Carcinoma Presenting as an Intracardiac Tumour Without Involving the Inferior Vena Cava

**DOI:** 10.7759/cureus.48679

**Published:** 2023-11-12

**Authors:** Neeraj Joshi, Shahnawaz Hashmi, Zulqarnain Afzal, Iwan Harries

**Affiliations:** 1 Cardiology, University Hospitals Bristol and Weston NHS Foundation Trust, Weston-Super-Mare, GBR; 2 Internal Medicine, University Hospitals Bristol and Weston NHS Foundation Trust, Weston-Super-Mare, GBR; 3 Cardiology, University Hospitals Bristol and Weston NHS Foundation Trust, Bristol, GBR

**Keywords:** transthoracic echocardiography, abdomen and pelvis, ct chest, cardiac mri (cmr), inferior vena cava (ivc), cardiac metastasis, renal cell carcinoma (rcc)

## Abstract

Renal cell carcinoma (RCC) is an aggressive tumour, with 25% of the cases presenting with distant metastases at the time of diagnosis. Approximately 33% of the patients with RCC eventually develop metastatic spread. RCC can metastasize to various sites including the lung, liver, bone, brain, adrenal gland, and more. Cardiac metastasis is rare in RCC, but even rarer in the absence of inferior vena cava (IVC) involvement. This case report presents a 60-year-old male patient who was referred by his general practitioner due to breathing difficulties. An initial echocardiogram revealed a right ventricular outflow tract obstruction caused by a mass. A subsequent cardiac MRI showed a right ventricular mass with features suggestive of a metastatic spread. A CT scan of the thorax, abdomen and pelvis was done to ascertain the primary tumour which revealed RCC, without involving the IVC. Due to the presence of metastases, advanced disease, and heavy tumour burden, the multidisciplinary team concluded that there were almost negligible treatment options available at that stage and recommended the best supportive care and community hospice support. The patient was discharged once his symptoms improved, as per his request, and he passed away peacefully at home within a month. This case highlights the very rare occurrence of cardiac metastasis of RCC without IVC involvement. It also illustrates the approach and investigations involved in the evaluation of complex cardiac masses.

## Introduction

Renal cancer is among the most common cancers in the United Kingdom (UK) and generally occurs in adults in their 60s or 70s, though rarely seen under 50s as well. Every year over 12,500 people in the UK are diagnosed with this condition [1]. Renal cancer usually affects only one kidney, and it is rare for it to present bilaterally. Although there are different types, about 80% of kidney cancers are renal cell carcinomas (RCCs). Metastatic renal cell carcinoma (mRCC) carries a grim prognosis, as the median survival time typically ranges from a mere six to 12 months, with a two-year survival rate falling between 10% and 20% [2]. Frequent sites of metastasis include the lung, liver, bone, brain, and adrenal gland. Case reports have illustrated the ability of RCC to appear in diverse locations throughout the body, with hematogenous spread being the most common mode of dissemination [2]. Cardiac metastasis of RCC is common as well but it almost always involves the inferior vena cava (IVC) in such cases. When there is no involvement of the IVC, cardiac metastases are exceedingly uncommon in patients with mRCC. In this case, we describe a scenario of a patient whose RCC was diagnosed after the patient was sent to the cardiac clinic of our hospital by their general practitioner (GP) for evaluation of recent-onset shortness of breath (SOB).

## Case presentation

**Figure 1:**
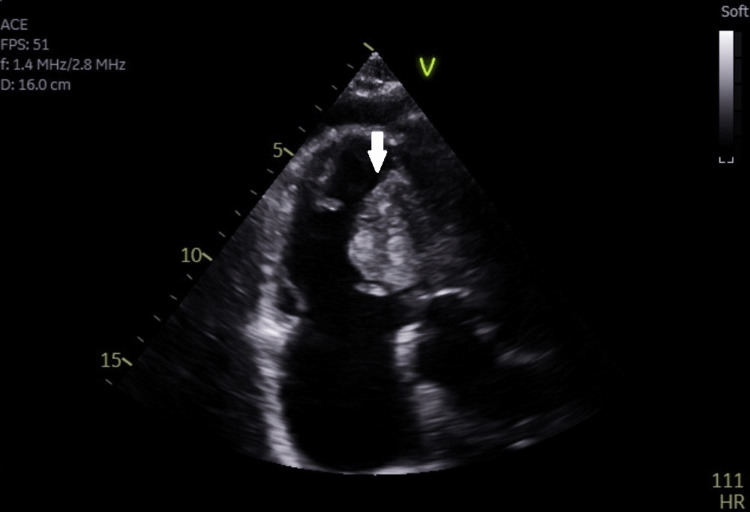
Note the large mass (arrow) adjacent to the anterior free wall with right ventricular outflow tract obstruction.

We describe the case of a 60-year-old male who was referred to our cardiology outpatient clinic by his GP for evaluation of his recent onset SOB. The GP had ruled out respiratory causes of said SOB and had referred the patient for an echocardiography scan. Except for bilateral pedal oedema and a tricuspid regurgitation murmur, the rest of his examination was reassuring. A routine ECG done before the echocardiography showed an S1Q3T3 pattern, which is sometimes suggestive of a pulmonary embolism (PE). Echocardiography was done immediately, which showed a dilated right ventricle (RV) with impaired systolic function, especially mid to apical radial function. RV hypertrophy was also seen with a free wall measured up to approximately 8 mm. A large echo-dense mass of irregular shape and minimal mobility was seen adjacent to the anterior free wall with right ventricular outflow tract (RVOT) obstruction. Small localised pericardial effusion was also seen adjacent to the RV free wall (Figure 1).

**Figure 2:**
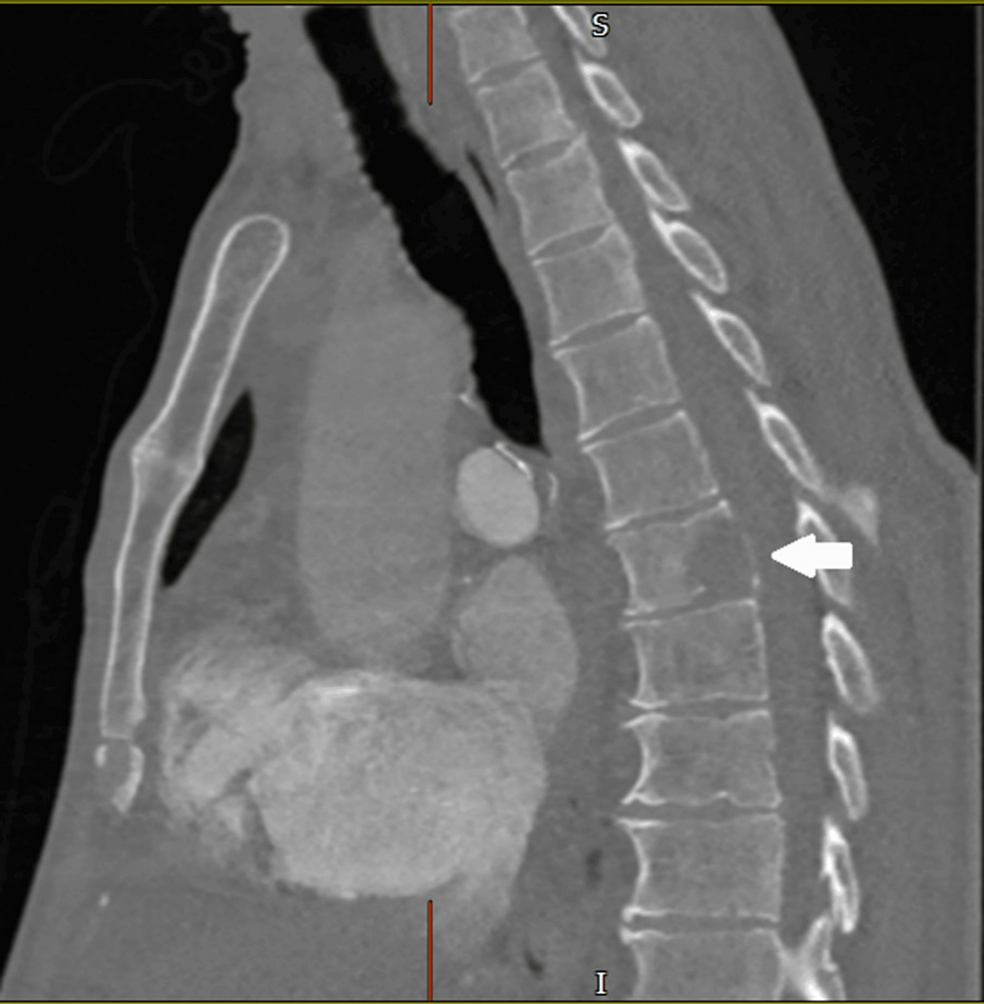
Note the lytic lesion at the T7 vertebra (arrow).

The patient was admitted directly via the outpatient department under cardiology. A CT pulmonary angiogram (CTPA) was scheduled immediately, which showed sufficient small filling defects in the sub-segmental branches of the pulmonary artery in the right lower lobe, suggesting PE. The radiologist also made note of a large obstructing thrombus in the RV, which was not extending into the pulmonary trunk. No evidence of acute heart strain was found; however, mild pericardial effusion was seen. Thus, all the findings of our echocardiography report were corroborated. A special feature of this CT scan was that the radiologist added a note at the end about a large lytic lesion present in the posterior part of the T7 vertebral body with extension into the vertebral arch, which he thought was probably metastatic (Figure 2).

**Figure 3:**
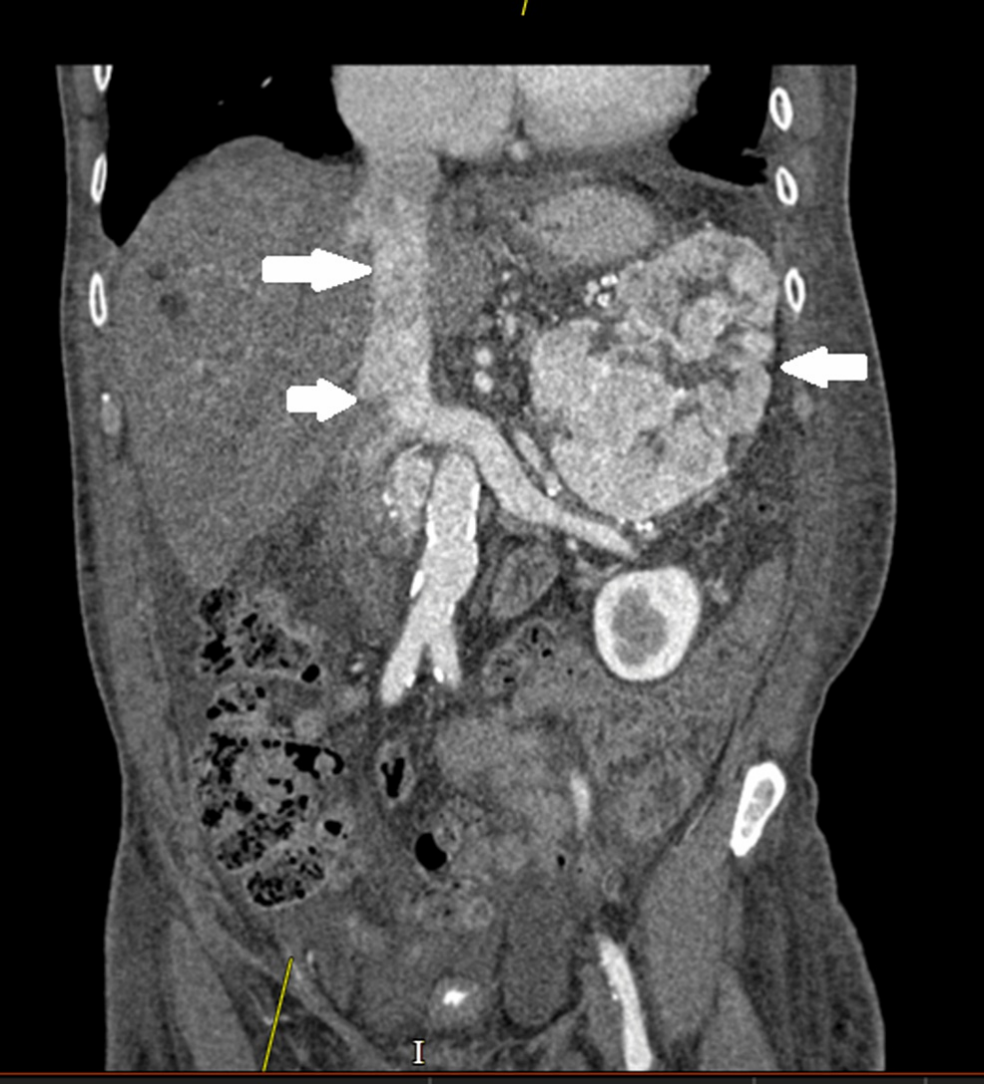
Note the L kidney mass (arrow on right) and that the inferior vena cava (arrows on the left) appears thrombus/tumour-free.

In light of these findings, a CT scan of the abdomen and pelvis (CTAP) with contrast was done to try to locate the malignancy. CT showed a large 12.5 x 12.4 x 8.3 cm enhancing mass with central hypoattenuation centred on the upper pole of the left kidney (Figure 3). The left renal veins were opacified normally with no evidence of tumour thrombus seen. Similarly, the IVC and the right kidney appeared normal. Aortocaval nodal metastases as well as enlarged para-aortic nodes in the upper abdomen were seen as well (Figure 3).

**Figure 4:**
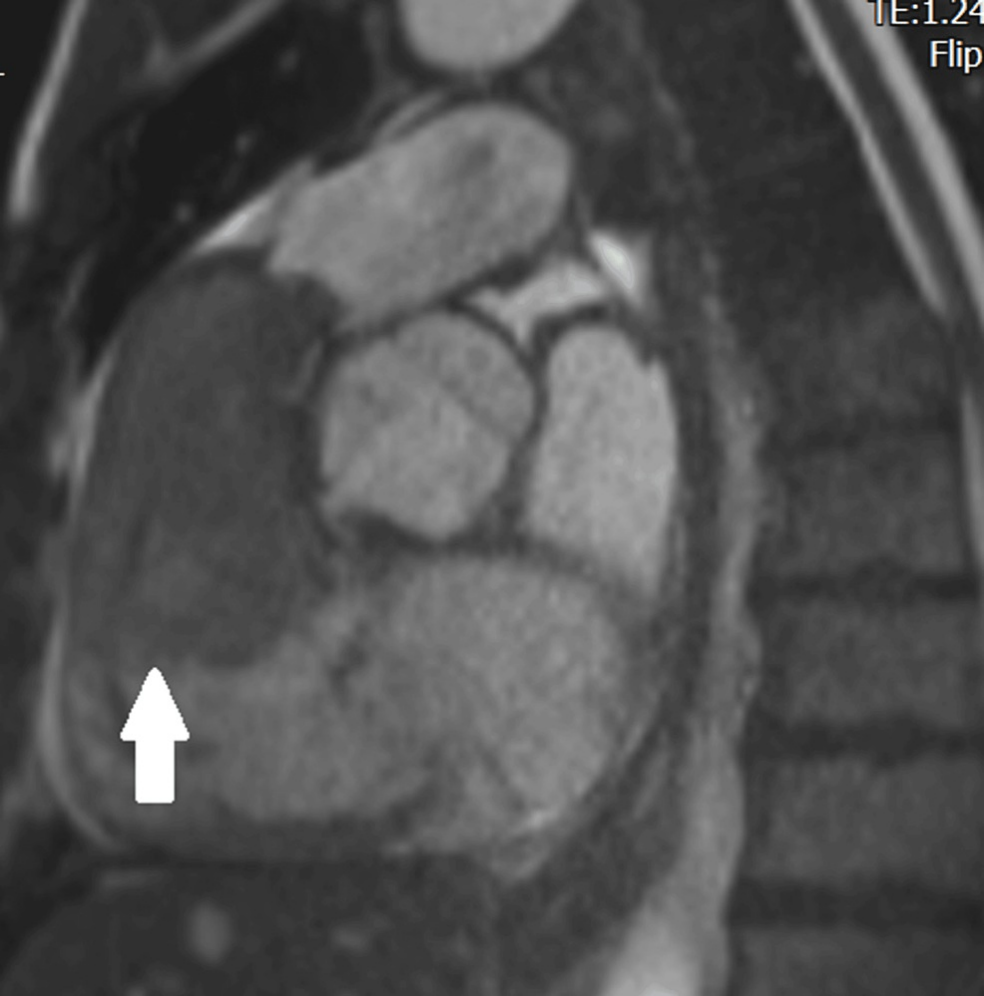
Note the bulky right ventricular mass that is causing the right ventricular outflow tract obstruction obstruction.

**Figure 5:**
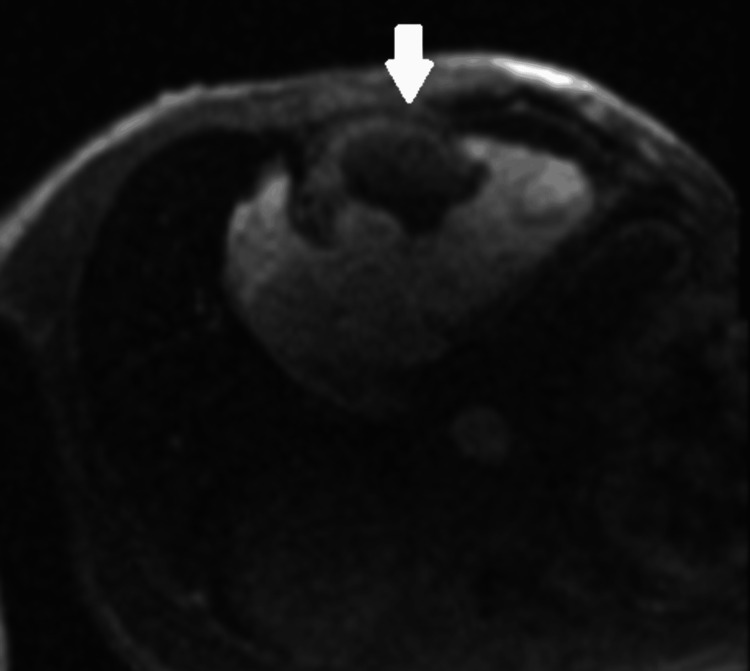
Perfusion scan in cardiac MRI showed subtle heterogeneous enhancements, highly favouring the diagnosis of metastases rather than thrombus.

Based on all these findings, a diagnosis of mRCC was made. The case was then discussed with our hospital’s parent tertiary cardiology department after starting therapeutic anticoagulation and diuretics, who advised the transfer of the patient. Given that it is unheard of for an intracardiac tumour thrombus extension from RCC without involving IVC, it was decided to perform a cardiac MRI scan to determine the morphology of the lesion inside the heart. Cardiac MRI showed bulky RV mass associated with RVOT, RV dilatation, severe RV impairment, severe tricuspid regurgitation and cardiac decompensation (Figure 4). A special note was made in the absence of the IVC tumour/thrombus. Characteristics of the mass on enhancement studies favoured tumour because it showed foci of patchy uptake, but had some associated superadded thrombus formation. T1-weighted images showed hypointensity, T2-weighted images showed hyperintensity on fast imaging with steady-state free precession, while a perfusion scan showed subtle heterogeneous enhancements. All these characteristics highly favoured the diagnosis of metastases, rather than thrombus (Figure 5).

Because of the severe disease burden and its widespread nature, it was decided in the multidisciplinary team meeting that the patient was not a surgical candidate nor would he benefit from complex systemic therapy. Detailed conversations were held with the family and patient, who wished for palliative care only and did not wish for any resuscitation. The palliative care team was taken on board for “end-of-life” care and the bereavement office was contacted for psychosocial support due to the suddenness of the disease. The patient was managed symptomatically, and after some improvement, he wished to go home. Given that he had capacity, he was discharged home with hospice community care support. He remained at home and peacefully passed away after one month.

## Discussion

RCC has various subtypes, with the most prevalent one being clear-cell renal cancer. Less frequently encountered variants include papillary RCC and chromophobe RCC. When detected in its early stages, RCC is often curable, but the likelihood of a cure diminishes significantly once metastasis occurs. RCC is characterized by a considerable propensity for metastasis, with approximately 20-30% of patients presenting with metastatic or locally advanced disease at the time of diagnosis [3]. The patterns of metastases are not clearly defined; hence, one can see patients with metastasis to unusual sites at the time of diagnosis of RCC [4]. Approximately 33% of individuals diagnosed with RCC develop metastatic spread. The treatment of patients with mRCC is challenging due to the limited effectiveness of existing therapeutic options [2]. In many cases, there are no obvious symptoms at first and kidney cancer may be found only during tests for another condition or reason. Unusual sites of metastases can have a substantial impact on the treatment and prognosis of patients with mRCC. RCC usually spreads through the haematogenous route, mainly involving the brain, bone, liver, adrenals and lungs; however, there are cases of it involving almost every organ.

RCC has a high inclination towards venous invasion. RCC can invade through the renal vein into the IVC, and can then extend into the lumen, with tumour-thrombus complex formation occurring in about 5% to 15% of all cases. The tumour can sometimes extend right up to the right cardiac chambers as well from there in about 1% of the cases [5]. There are four stages of this tumour thrombus extension to the heart; type I involves the intravascular tumour reaching the renal vein but not the IVC, type II involves the IVC being occupied up to the level of the hepatic veins, while in types III and IV the IVC above the diaphragm and, subsequently, the right cardiac chamber are involved [6]. In almost all of the cases, this spread to the right heart chambers occurs via the IVC. It is extremely rare for RCC to cause tumour thrombus extension to the right heart without involving the IVC.

While primary cardiac tumours are rare, cardiac metastases are common and represent up to 9% of the intracardiac masses. These are a result of either direct extension or haematogenous or lymphatic spread [7]. Here, we present a case of an RV tumour without involvement of the IVC or renal veins, which is extremely rare. In a retrospective analysis that combined data from four clinical trials involving 1,765 patients with mRCC, it was observed that intracardiac metastases without any involvement of the IVC occurred in less than 1% of cases, specifically in 10 individuals [8]. In this case, the patient had been referred by the GP for evaluation of his ongoing SOB which was non-respiratory in nature. Echocardiography revealed an RVOT obstruction due to a mass, which was later confirmed as a thrombus on a subsequent CTPA. CT of the abdomen and pelvis with contrast showed no tumour thrombus in either renal veins or IVC. Subsequent cardiac MRI showed bulky RV mass associated with RVOT, RV dilatation, severe RV impairment, severe TR, and cardiac decompensation. A special note was made of the absence of IVC tumour/thrombus. Characteristics of the mass on enhancement studies favoured tumour because it showed foci of patchy uptake, but had some associated superadded thrombus formation.

On a literature review, although there are some cases of intracardiac tumour metastases without IVC or renal vein involvement such as those reported by Raiker et al. [7], Ansari et al. [9], Briasoulis et al. [10], and Zustovich et al. [11], it still remains a relatively rare scenario. In cases where intracardiac metastases are suspected in patients, the initial recommended imaging test is transthoracic echocardiography, which is used to identify the location, size, and mobility of cardiac lesions. Nonetheless, it is important to note that echocardiography has its limitations, particularly when assessing patients with suboptimal acoustic windows and evaluating extracardiac structures [12]. The gold standard for diagnosing intracardiac metastases is tissue biopsy and histology but given that RCC lesions are usually very vascular, this procedure carries significant mortality and morbidity risks. Cardiac MRI is a non-invasive, sensitive test and can help with confirming the diagnosis of metastases. Therefore, we used cardiac MRI as well in our case for confirming the diagnosis.

Every RCC patient should ideally begin treatment with an anti-programmed death-1-based therapy, according to the Society for Cancer Immunotherapy. However, there is not enough data to determine the effectiveness and safety of checkpoint inhibitors for patients with uncommon metastatic sites, such as intracardiac metastases like in our case. Cardiac involvement without IVC tumour is not that common and presents a unique treatment challenge, even with the new anti-programmed cell death receptor-1 monoclonal antibody [13]. Additionally, surgical treatment of RCC with atriocaval involvement has significant perioperative mortality and complication rates [14]. Generally speaking, mRCC with intracardiac spread has a dismal outcome and poor prognosis. Patients frequently present late when the disease has progressed significantly. Our patient had a very high tumour burden on presentation and was not a surgical patient, hence, without treatment options, was discharged home once his symptoms improved.

## Conclusions

Patients with mRCC and intracardiac metastases, in the absence of IVC involvement, are exceptionally rare. Generally, this subpopulation experiences unfavourable outcomes as they are often diagnosed at an advanced clinical stage. The optimal treatment approach for RCC patients with cardiac metastases remains uncertain. Intracardiac metastases can manifest with various clinical consequences, including haemodynamic disturbances from ventricular outflow obstruction, embolism, and cardiac dysfunction, or sometimes with seemingly minor symptoms such as increased breathlessness. Given the non-specific nature of these symptoms, it is imperative to maintain a high clinical suspicion to guide appropriate investigations. Echocardiography, though valuable, may have limitations in distinguishing cardiac metastases from thrombi, endocarditis, or primary tumours. Therefore, additional cardiac MRI or PET/CT imaging is often necessary for precise differentiation and to inform treatment decisions.
